# Role of Myokines in Myositis Pathogenesis and Their Potential to be New Therapeutic Targets in Idiopathic Inflammatory Myopathies

**DOI:** 10.1155/2020/9079083

**Published:** 2020-07-24

**Authors:** Vlad Mageriu, Emilia Manole, Alexandra E. Bastian, Florica Staniceanu

**Affiliations:** ^1^National Institute of Pathology, Bucharest, Romania; ^2^Medlife SA, Bucharest, Romania; ^3^Colentina Clinical Hospital, Research Center, Pathology Department, Bucharest, Romania; ^4^University of Medicine and Pharmacy, Bucharest, Romania; ^5^Colentina Clinical Hospital, Pathology Department, Bucharest, Romania

## Abstract

Idiopathic inflammatory myopathies (IIM) represent a heterogeneous group of autoimmune diseases whose treatment is often a challenge. Many patients, even after immunosuppressive therapy, do not respond to treatment, so new alternatives have been sought for this. Therefore, other signaling pathways that could contribute to the pathogenesis of myositis have been investigated, such as the expression of myokines in skeletal muscle in response to the inflammatory process. In this review, we will refer to these muscle cytokines that are overexpressed or downregulated in skeletal muscle in patients with various forms of IIM, thus being able to contribute to the maintenance of the autoimmune process. Some muscle cytokines, through their antagonistic action, may be a helpful contributor to the disease modulation, and thus, they could represent personalized treatment targets. Here, we consider the main myokines involved in the pathogenesis of myositis, expressing our view on the possibility of using them as potential therapeutic targets: interleukins IL-6, IL-15, and IL-18; chemokines CXCL10, CCL2, CCL3, CCL4, CCL5, and CCL20; myostatin; follistatin; decorin; osteonectin; and insulin-like 6. An interesting topic regarding the complex connection between myokines and noninflammatory pathways implied in IIM has also been briefly described, because it is an important scientific approach to the pathogenesis of IIM and can be a therapeutic alternative to be considered, especially for the patients who do not respond to immunosuppressive treatment.

## 1. Introduction

This review aims to examine the most recent studies regarding the presence and role of myokines in inflammatory myopathies. There are few reviews concerning the activity and role of myokines in normal muscle and in other muscular pathologies, but from our knowledge, there is no recent review specifically dedicated to myokines in myositis. In addition, we would like to focus the attention on myokines as possible therapeutic targets in idiopathic inflammatory myopathies (IIM), as there are still difficulties in treatment approaches that do not have the expected results yet. In this complex group of diseases, there are overlapped clinical diagnostics, nonresponder patients, or a complicated pathogenesis not elucidated so far. Having a summary of the recent studies and an overview of possible further research, readers can easily draw conclusions on the results already achieved and some starting points for investigations to be made in this field.

Myositis or IIM represent a heterogeneous group of autoimmune diseases characterized by muscle weakness, the presence of inflammatory muscle infiltrates, as well as overexpression of MHC class I in muscle fibers sarcolemma. IIM are traditionally divided into 5 subtypes: polymyositis (PM), dermatomyositis (DM), inclusion body myositis (IBM), autoimmune necrotizing myopathy, myositis overlap-syndromes, e.g., those associated with cancers or other systemic autoimmune diseases [1]. The myositis classification is constantly changed. The classification criteria have been a subject of debate for many years, but the Bohan and Peter criteria have remained the basics [2, 3], valid today, but to which other categories have been added over the years. Most of the modifications refer to the clinico-serological criteria by the discovery of new myositis-specific and myositis-associated autoantibodies. A recent review regarding the new classification criteria of myositis is that of Leclair and Lundberg [4]. In these conditions, sometimes it is difficult to put a diagnosis because of heterogeneity presented by this group of diseases, and also because of overlapping syndromes. In addition to clinical examination, laboratory tests, MRI investigation, muscle and skin biopsy, and electromyography are performed.

Inflammation is constantly present, but there are clear differences between the IIM forms: macrophages, CD8+ T-cells, mainly involved in PM and IBM, and CD4+ T-cells and B-cells, mainly involved in DM. In DM, inflammatory infiltrates are found especially around blood vessels, while in PM and IBM, it is an inflammatory cell invasion of nonnecrotic fibers.

Other morphological changes in skeletal muscles are muscular atrophy, the presence of necrotic fibers, collagen proliferation, and rimmed vacuoles (in IBM). An overexpression of MHC class I and MHC class II in sarcolemma are present in all types of IIM [5–7]. We mentioned all these pathological changes observed in the muscles, because they are related to the subject presented in this review.

Besides the fact that the diagnosis is sometimes difficult to establish, it is also noted that anti-inflammatory treatment often does not give the expected results, and muscle weakness persists.

In recent years, a special attention has begun to be given to skeletal muscle cytokines called myokines. They are involved in the inflammatory process triggered by the immune system, aggravating or ameliorating inflammatory pathology. Thus, myokines may become important therapeutic targets for patients with IIM. Furthermore, the presence of myokines in muscle biopsy or in blood samples of IIM patients could be an indication for a specific and personalized diagnosis.

Given the fact that myositis is such a heterogeneous group of autoimmune diseases, it is very important to highlight new data on this pathology, both for a correct diagnosis and for finding new methods of treatment. In this review, we aim to draw attention to the role of myokines in IIM pathology, as well as to the existence of complex interconnected cellular signaling pathways in which muscle cytokines play an increasingly important role.

## 2. Myokines Definition

Myokines are cytokines, chemokines, or peptides produced and released by muscle cells [8] under the action of contractile activity [9] or as an effect of other stimuli, just like an endocrine organ, as described in recent years [10]. Cytokines are a large group of small proteins whose primary role is associated with immune response. They may have proinflammatory and anti-inflammatory functions, depending on the initial stimuli, target cell, or additional cytokines released in conjunction [11].

Myokines have begun to be more and more studied in connection with muscular pathology such as cancer cachexia [12], muscular dystrophy [13–15], and myositis [16]. There are myokines that have positive effects, others that have negative effects, and some that have both positive and negative effects, depending on muscle pathology or metabolic conditions.

## 3. Role of Myokines in Idiopathic Inflammatory Myopathies

There are studies that have shown that myokines can have a pathogenic effect in the skeletal muscles, can induce muscle weakness, and decrease muscle mass in myopathies, both in muscular dystrophies and myositis as well as in muscular cachexia from cancers.

**Figure 1:**
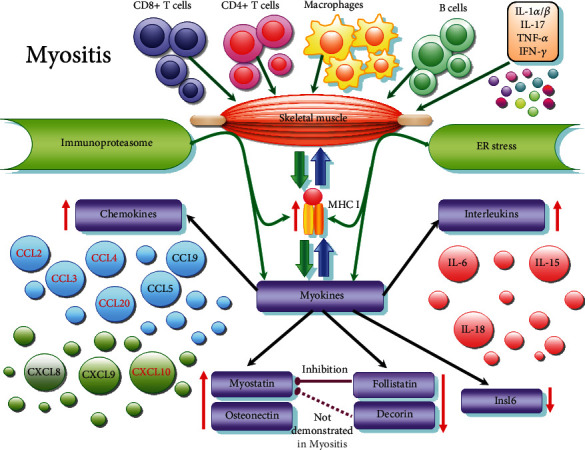
Schematic representation of myokines released in myositis. Skeletal muscle inflammation is induced by invading immune cells (thin dark green arrows): macrophages, T-cells (CD4 + T-cells, CD8 + T-cells), B cells which release proinflammatory cytokines (IL: interleukin; IFN: interferon; TNF: tumour necrosis factor). Most important cytokines related to myokines releasing are IL-1*α*/*β*, IL17, TNF-*α*, and INF-*γ*. In response to inflammation, MHC class I is overexpressed on the sarcolemma, contributing to muscle injury. Moreover, MHC I activates ER stress response and inflammasome activation. Immunoproteasome and endoplasmic reticulum (ER) stress contribute, in turn, to MHC I overexpression (thin green arrows). Under these conditions, muscle cells secrete myokines (thick green arrows), such as interleukins—IL-6, IL-15, IL-18; chemokines—CCL2, CCL3, CCL4, CCL5, CCL9, CCL20, CXCL8, CXCL9, CXCL10 (the most important in red); myostatin, follistatin, osteonectin, decorin (probably present, but uninvestigated in myositis), and insulin-like 6 (Insl6). Myokines, in turn, attract inflammatory cells, maintaining, to a certain extent, inflammation (thick blue arrows).

IIM are autoimmune diseases, cytokines released by inflammatory infiltrate cells in muscle tissue inducing the production and releasing of myokines and overexpression of MHC I [17–19]. Myoblasts, in addition to the production of chemokines, may be involved themselves in the recruitment of leukocytes [17, 20]. Once released, myokines, interacting with the endoplasmic reticulum stress pathways, can activate the production of inflammatory cytokines, helping to maintain inflammation on the one hand, and maintaining muscle weakness in the absence of inflammation on the other hand [21, 22] (Figure 1). Contrariwise, there are studies that have clearly shown that endurance exercise, causing the release of myokines, is a successful therapeutic intervention in IIM [23].

In this review, we will refer to the role of myokines in myositis (Table 1). It is important to know their mode of action in certain physiological or pathological conditions, so that the modulation of their expression could become a therapeutic model. Controlling myokines in myositis may become an important factor in finding an appropriate or personalized treatment.

Among the myokines that have been cited in the literature as having a possible role in modulating the pathogenesis of myositis, we will address the following: interleukins (IL-6, IL-15, IL-18), chemokines (CXCL10, CCL2, CCL4, CCL5, CCL20), myostatin, follistatin, decorin, osteonectin, and insulin-like 6.

## 4. Interleukins

Interleukins (ILs) are cytokines that were first seen to be expressed by leukocytes, hence the name. But later, they have been found to be produced by many other types of cells. ILs are key factors in the functioning of the immune system, in activation and differentiation of immune cells by binding to high-affinity receptors on cell surfaces. They stimulate the development and differentiation of T and B lymphocytes and also of haematopoietic stem cells [24, 25]. ILs are involved in systemic inflammation and immune system modulation, so they play important roles in fighting infectious diseases or cancer.

There have been identified around 40 different ILs [26]. They are implied in multiple signaling pathways, exhibit diverse roles in immune regulation and cellular networks, and target numerous proteins that regulate biological responses. Identification of ILs antagonists (antibodies and receptor antagonists), as treatment option for autoimmune disorders and other inflammatory diseases, is a target in current and future research.

The best-known interleukins that are secreted by the skeletal muscle are IL-6, IL-15, and IL-18 which are presented below.

### 4.1. IL-6

IL-6, a glycoprotein of 21-28 kDa, is a pleiotropic prototypical cytokine (four-helix bundle cytokine) that activates an acute immune response in infections and in muscle tissue injuries [27, 28]. It is the most studied myokine in IIM. It acts as a mediator of innate and adaptive immune responses [27, 28]. IL-6 exerts both an endocrine and a metabolic function in several organs, such as the pancreas, gut, fat, liver, and skeletal muscle.

IL-6 was among the first myokines seen upregulated after intense exercise [29], up to 100-fold in relation to other cytokines such as TNF-*α* or IL-1*β* [30–32]. IL-6 upregulates antioxidant defenses as response to oxidative stress, as shown by Sacheck et al. [33].

The number of IL-6 receptors also increases after exercise [34]. Serrano et al. in an *in vitro* study on mice lacking IL-6 gene in skeletal muscle [35] have shown that this myokine is an important regulator of muscular hypertrophy mediated by the satellite cells in normal muscle.

Several studies have revealed that IL-6 has a dual role in muscle, in some pathological cases inducing atrophy [36, 37], in other cases supporting regeneration by increasing myoblasts proliferation [38]. A review showing the pivotal role of IL-6 between skeletal muscle wasting and renewal belongs to Belizario et al. [39]. A surprising fact was observed concerning this dual effect of IL-6; although, this cytokine has a proinflammatory role in rheumatic diseases, for example [32], when released from muscle it has an anti-inflammatory role [40]. It was shown that the role of IL-6 is predominantly played in metabolism rather than in inflammation. IL-6 regulates energy metabolism. We consider that these results could have similar connotations in IIM, and further research is needed to define the role of IL-6 in this pathology.

Given the dual role of IL-6, in cachexia high plasma levels of this myokine cause a decrease in muscle weight [41], cause muscle atrophy [42] and an increased cathepsin level. Studies on a transgenic mouse model have shown that IL-6 is involved in the regulation of muscle protein degradation [36]. Some authors consider that the significance of IL-6 overexpression in skeletal muscle in cachexia [12] is the same as in the muscle with inflammatory pathology, increasing the disease progression.

Bilgic et al. [43] observed in patients with DM that there is a correlation between IL-6 serum levels and the disease activity, being a candidate biomarker for adult and juvenile DM.

An *in vivo* research on a murine model of PM showed that a treatment with anti-IL-6 receptors antibodies had a positive effect [44]. Experimental studies on human muscle cell cultures showed that IL-6 production in human myoblasts is induced by the presence of TNF-*α*, IL-17, and IL-1*β* [18, 45]. Thus, the muscle inflammation on isolated myoblasts was induced by incubation with these exogenous cytokines arguing for an increase in IL-6 levels in response to inflammation. As a result, blocking IL-6 signaling pathway could be a potential therapy in IIM. In fact, this blockage has been approved for treatment in rheumatoid arthritis [46].

Scuderi et al. showed on a transgenic mouse IL-6-/- treated with rabbit myosin to induce IIM, which there was no inflammation in the muscle fibers, a complete absence of necrosis and leukocyte infiltrates, as well as the absence of myofiber regeneration. Muscle infiltrates present in muscle were macrophages. Their conclusion was that the absence of IL-6 prevents the infiltration of muscle with monocytes, due to a chemotaxic inhibition [47].

A study on patients with IIM, made by Loaiza-Felix et al. [48] to assess whether levels of serum adipokines may be disease markers, showed higher levels of IL-6 in patients compared with healthy individuals. It is interesting that in this study IL-6 is considered as adipokine, cytokine secreted by adipose tissue. High serum IL-6 levels were also found in IIM patients with interstitial lung disease [49]. Given that IL-6 is both adipokine and myokine, it can be called adipo-myokine. Even though their function has not yet been completely clarified, adipo-myokines seem to have a double effect, beneficial or adverse, depending on the extent and kinetics of these molecules in serum [50, 51].

In conclusion, the role of IL-6 in myositis is not yet fully understood, and further studies on this cytokine as a therapeutic target should be considered, taking into account both the role of IL-6 as myokine, but also as a serum cytokine, or its dual role played in metabolism.

### 4.2. IL-15

IL-15 is a 14-15 kDa molecule, member of the 4*α*-helix bundle cytokine family. It is another myokine released by the muscle after exercise.

IL-15 and its receptor, IL-15R*α*, have widespread expression [52, 53] at the transcriptional level in many normal human tissues and cells including skeletal muscle [52], but they are rarely detected at the protein level under normal conditions [54].

Initial research concerning the functionality of IL-15 appeared to indicate a role in skeletal muscle hypertrophy [55]. However, more recent studies have indicated a potential alternative role in muscle oxidative and fatigability properties [56, 57]. IL-15 stimulates myosin-heavy chain accumulation in differentiated myocytes [55, 58].

Concerning the IL-15 expression in response to different resistance training exercises, previous literature has reported a variability in its serum levels. Riechman et al. [59] observed a statistically significant increase in plasma IL-15 following a resistance training regimen. In contrast, Nielsen et al. did not observe any change in plasma IL-15 following a resistance exercise protocol [60].

In myositis, a report based on patients with PM and DM [61, 62] showed an upregulation of IL-15 in muscle fibers and serum, in correlation with disease severity. It was suggested a possible role of IL-15 in the PM and DM mechanisms. IL-15 production is stimulated by IL-1*α*/*β*, TNF-*α*, IFN-*γ*. and LPS, and also by CD40 ligation. In turn, IL-15 stimulates the dendritic cells, NK cells, and effector T-cells [63].

Zhong et al. investigated the IL-15 and IL-15R*α* expression in skeletal muscle of patients with PM and DM after treatment [64], and they observed that IL-15/IL-15R*α* expression was correlated with clinical findings: patients with a high number of IL-15-expressing cells in muscle tissue after immunosuppressive treatment had less improvement in muscle performance, which might indicate that IL-15 has a role in causing muscle weakness.

Another research on PM patients and experimentally, on a PM rat model [65], reported that the levels of CD163 macrophages were dramatically reduced after the treatment with the antibody anti-IL-15, indicating that IL-15 is closely connected to CD163 macrophages and has a significant influence upon the pathogenesis of idiopathic myositis. The authors showed also that being involved in the NF-*κ*B signaling pathway, IL-15 can upregulate the expression levels of matrix metallopeptidase 9 (MMP-9). MMP-9 has been proved to be involved in the inflammatory process of muscle degeneration, and there may be an association between MMP-9 and the emergence of PM [66]. The researchers' opinion was that IL-15, as a key regulator in PM, is likely to become a promising therapeutic target and a possible treatment for multiple myositis.

Po-Lin et al. [67] hypothesized that IL-15 contributes to the development of autoimmune inflammation in the myositis by promoting autoreactive CD8+ T-cell function, constituting a potential therapeutic target.

However, the exact role of IL-15 in myositis is not yet known enough, and further studies are needed to bring new data on this. However, it appears that elevated levels are harmful and help maintain inflammation in myositis.

### 4.3. IL-18

IL-18 is an immunoregulatory cytokine which induces interferon-gamma (IFN-*γ*) [68]. As a consequence, the first name of IL-18 was IFN-*γ*-inducing factor, changing in IL-18 after the molecular cloning [69]. The primary sources for active IL-18 are macrophages and dendritic cells, but its precursors are present in epithelial cells [70].

IL-18 is a proinflammatory cytokine and together with IL-12, induces cell-mediated immunity. As myokine, IL-18 plasma levels were found normally increased after acute exercise [71].

A study concerning the involvement of IL-18 in muscle pain suggests that increased neutrophil numbers and IL-18 secretion from neutrophils produce mechanical hyperalgesia induced by repeated excessive muscle contraction [72]. This research was based on the observations that neutrophils are a source of IL-18 [73, 74].

The activity of IL-18 is balanced by the naturally occurring IL-18 binding protein (IL-18BP), as a study has shown in humans [75], and local IL-18BP administration attenuated hyperalgesia caused by excessive muscle contraction [72].

IL-18 has been implicated in numerous disorders, including autoimmune diseases [75]. Helmers et al. reported that IL-18 expression was predominantly localized to inflammatory cells and capillaries in patients with myositis compared with healthy controls where IL-18 was found mostly in capillaries. They observed also that the total IL-18 expression appeared lower in biopsies from patients receiving and improving with immunosuppressive treatment [76].

Gono et al. [77] observed that serum IL-18 level in patients with DM and PM was higher compared with healthy controls. Moreover, patients with DM had higher levels than PM patients, and DM patients with interstitial lung disease (ILD) had higher IL-18 serum levels than DM patients without ILD. In conclusion, serum IL-18 was strikingly elevated in DM patients, associated particularly with disease activity and ILD complications. A similar study was conducted by Yang et al. [78], showing the same results for DM–DM with ILD; however, no higher serum IL-18 levels were observed in patients with PM compared with controls.

The studies on IL-18 expression in IIM are few, but the important results obtained so far necessitate future investigations. It is clear that elevated levels of IL-18 in IIM may be a diagnostic marker and may be a therapeutic target.

## 5. Chemokines Family

Chemokines are small cytokines with a chemoattractant role, which guide the migration of leukocytes into the area of inflammation and regulate the homeostatic trafficking of lymphocytes and dendritic cells. Functionally, they are divided into 2 groups: homeostatic (responsible for basal leukocytes migration) [79] and proinflammatory [80]. The latter are produced in pathological conditions, under the action of proinflammatory stimuli, such as IL-1, TNF-*α*, and INF-*γ*, active in myositis. The chemokines detected in IIM muscle are CCL2, CCL3, CCL5, CCL9, CXCL8, CXCL9, and CXCL10 [81–83].

Accumulation of chemokines in muscle may enhance the activation and migration of leukocytes, maintaining autoimmune attack. There are *in vitro* studies, on myoblast cultures, that have shown that proinflammatory stimuli can induce the synthesis of chemokines in muscle cells, thus helping to perpetuate inflammation [20].

### 5.1. CXCL10 (C-X-C Motif Chemokine 10)

CXCL10, known also as interferon gamma-induced protein 10, has been defined as a chemokine that plays a role in the immunopathogenesis of autoimmune diseases, so in IIM, through the initiation and maintenance of type 1 T-helper cells (Th1) response [84]. It is an 8.7 kDa protein belonging to CXC chemokine family.

In a CIM (C-protein-induced myositis) mouse model, Jinhyun et al. [85] have shown that the expression of CXCL10 and its receptor CXCR3 (expressed on Th1 cells) was increased. Its blocking with anti-CXCL10 antibody or anti-RVG1 (rotavirus IgG1) suppressed muscle inflammation.

A study on patients with sporadic IBM (sIBM) revealed increased levels of CXCL10, and also CXCL9, in serum and muscle lysates [86]. However, the authors have shown that these results are not specific for sIBM and may be valid for other types of myositis also.

De Paepe et al. [87], in a study on patients with different forms of myositis, reported an abundant expression of CXCL10 both in T-cells and macrophages infiltrates in endomysium, in PM and sIBM, and also in T-cell aggregates present in perimysium, in DM. Concerning the CXCL10 receptor, CXCR3, the authors found it abundantly expressed in IIM, especially in PM and DM, on Th1 cells.

A research paper demonstrated that the levels of CXCL10 together with tumour necrosis factor receptor type II (TNFRII) and galectin 9 were increased in patients with active juvenile DM [88]. The results of this study revealed the CXCL10 as an important chemoattractant for monocytes, dendritic cells, and T-cells, which are present in the biopsy specimens of myositis patients and in their plasma also. These outcomes suggested that CXCL10 might also be a therapeutic target in patients with juvenile DM. In connection with these data, a human anti-CXCL10 antibody (MDX1100) was tested in clinical trials for ulcerative colitis and rheumatoid arthritis [89]. Other studies have suggested also the CXCL10 to be a therapeutic target in adult myositis patients [90].

Given these reports, which all showed an abundant expression of this chemokine and its receptor, we consider CXCL10 and CXCR3 receptor to be potential targets for a selective therapy in some forms of myositis.

### 5.2. CCL2, CCL3, CCL4, CCL5 (Chemokine C-C Motif Ligand 2, -3, -4, -5)

CCL2, CCL3, CCL4, and CCL5 are chemokines implied in IIM as chemotactic factors that coordinate the recruitment of leukocytes. It has been observed that MHC I overexpression in the muscle leads to the release of CCL chemokines. Thus, Lightfoot et al. [91] reported in an *in vitro* study on C2C12 myotubes transfected with a MHC I (H-2 kb) overexpression vector, using Lipofectamine2000, the release of CCL2, CCL4, and CCL5, together with IL-6, via the ER stress pathway.

A study on patients with IIM [48] have shown that serum CCL2 titres correlate strongly with CK levels, being expressed in macrophages and T-cells which actively invade muscle fibers [82]. An increased serum CCL2 levels were demonstrated in patients with juvenile DM, their concentrations being correlated with disease duration but not with disease activity [92].

Civatte et al. [93] have reported a strong expression of CCL3 and CCL4 in all IIM subtypes, revealed by RT-PCR and immunohistochemistry techniques. CCL4 was present in all vessels in DM, but in PM and sIMB, it was restricted to vessels in the vicinity of inflammatory exudates. CCL5 had a low expression in a few inflammatory cells. The CCR1 receptor expression was restricted to macrophages and s-IBM endothelial cells, whereas CCR5 receptor expression was observed in inflammatory cells invading nonnecrotic muscle fibers. The conclusion was that the upregulation of these myokines and some of their receptors may contribute to chronic inflammation in IIM. These results are confirmed by other authors too [81, 82, 94].

The CCL2 receptor, CCR2, was also found upregulated in IIM [95].

Therefore, it is likely that a better understanding of the molecular events leading to the formation of inflammatory infiltrates in IIM as well as a better description of various beta chemokines, and receptors will offer hope for a more selective immunotherapy in the future.

### 5.3. CCL20 (Chemokine C-C Motif Ligand 20)

It is a small cytokine of CC chemokine family, heaving chemotactic action for lymphocytes, named also MIP-3a (macrophage inflammatory protein-3a).

CCL20/MIP-3ais a chemokine involved also in dendritic cells migration [96]. There are studies that have shown that CCL20 induced migration of immature DC, of natural killer cells and T-cells also, but not of monocytes [97, 98].

Chevrel et al. [45] have revealed in an *in vitro* study (on primary cell culture from normal human muscle incubated with T-cell-derived cytokine IL-17 and monocyte-derived cytokine IL-1*β*) that low levels of a combination of IL-17 and IL-1*β* in muscle cells (human myoblasts) can trigger the expression/production of inflammatory factors, as IL-6 and the upregulation of CCL20/MIP-3a. They showed that IL-17 and CCL20 were immunohistochemically located in T-lymphocyte-rich areas in biopsies from patients with DM and PM. In addition to the attention paid to CCL20, the authors of the study considered IL-17 produced by lymphocytes in the myositis muscle (in PM and DM) as a possible therapeutic target.

Taking into account these reports, we can say that CCL20, along with other chemokines and interleukins with which it interacts, could represent a possible target in the IIM therapeutic strategies.

## 6. Myostatin

Myostatin, also called growth differentiation factor 8/GDF8, is a protein belonging to the TGF-*β* (transforming growth factor-beta) superfamily that negatively regulates muscle growth during development and muscle mass in adulthood [99]. In knocking out mouse models for myostatin gene, the muscle mass is larger [99].

Myostatin is synthesized as a precursor protein, and the processing action from myostatin precursor protein to myostatin has been proposed to occur intracellularly, possibly through the action of furin [100]. Outside the muscle fiber, myostatin is inactivated by its binding to follistatin, which thus inhibits its binding to cellular receptors on muscle fibers [99, 101].

A mode of action of myostatin has been proposed as follows: myostatin and activin A bind type 2 activin receptors. After dimerization, they bind activin receptor type 1 (ALK4/5) and signal through a TGF-*β* signaling pathway involving Smad2 and 3 phosphorylation [102]. Phosphorylation of Smad2 and 3 results in the downregulation of genes associated with muscle differentiation and inhibits AKT (protein kinase B) signaling. This AKT pathway is normally activated during muscle hypertrophy and often inhibited over muscle atrophy [103].

Myostatin inhibition has been shown to be beneficial in increasing of muscle mass [104, 105]. During the past 14 years in many clinical trials, attempts have been made to improve muscle function and muscle mass by inhibiting myostatin signaling pathway, targeting pathologies such as different muscular dystrophies or IBM. The results were not always what it was expected [106]. Preventing or reversing muscle atrophy remains an unmet medical need. Most of the preclinical or clinical studies for muscle atrophy therapy include myostatin inhibitors. The data obtained by Pirruccello-Straub et al. demonstrated a novel approach to myostatin inhibition, targeting the prodomain to modulate the activation of the mature myostatin rather than by blocking receptor-ligand interactions which may develop cross-reactivity with other TGF-*β* growth factors [107].

There are not many studies focusing on myostatin in IIM, but the interest in this field is growing. Wojcik et al. demonstrated for the first time that within sIBM muscle fibers, myostatin/myostatin precursor accumulates and associates with aggregates containing Amyloid-beta (A*β*). The expressions of myostatin precursor protein and myostatin dimer are increased, and myostatin precursor protein binds A*β* [108]. Given these results, a therapeutic attempt in s-IBM may be the decreasing myostatin/myostatin precursor.

Myostatin signaling pathway was reported to be upregulated in IBM [109] and an assay to block it was performed through gene therapy using follistatin as a myostatin inhibitor.

Another study showed that resistance training with vascular occlusion in a patient with IBM led to an increase in muscle mass and strength [110, 111], the myostatin playing an essential role in this mechanism. The myostatin gene expression was attenuated, while the gene expression of myostatin endogenous inhibitors, as follistatin, follistatin-like 3, and SMAD-7, was upregulated.

Decorin, such as follistatin, is another myostatin inhibitor. Although not studied in IIM, it is known to be overexpressed after exercise in normal muscle [112]. Here, we just want to draw attention to it as a possible modulator of myostatin. Future research is needed to evaluate the role of decorin and follistatin in IIM and their use in therapy by modulating myostatin expression.

## 7. Follistatin

Follistatin is a glycoprotein expressed in all tissues in variable concentrations [113]. It is an inhibitor of myostatin, promoting muscle growth by binding to ActRIIB (activin A bound to the extracellular domain of a type II receptor). In this way, follistatin neutralises the effect of various members of TGF-*β* (transforming growth factor-*β*) superfamily, including myostatin and activin–inhibin complex [114, 115].

Being an inhibitor of myostatin, follistatin may be a therapeutically target in IIM. Myostatin inhibition through follistatin gene intervention was proposed. Thus, a single administration of a vector carrying the follistatin gene resulted in an increase in muscle mass and strength, with reduced fibrosis. The experimental *in vivo* research was done on a mouse model with muscular dystrophy [116]. We consider that it is also an approachable subject for further studies on therapy in the IIM.

A clinical trial of follistatin gene transfer to 6 patients with sIBM has already been done. The patients received an intramuscular injection in quadriceps muscles of both legs with an isoform of follistatin (FS344) using AAV1 vectors, in combination with exercise. Six months later, there was observed an improvement in muscle regeneration and a reduction in fibrosis [117].

Vernerova et al. reported higher follistatin and lower myostatin levels in circulation and attenuated expression of myostatin pathway signaling components in skeletal muscle in IIM patients compared with healthy controls [118].

There is little information regarding the potential pathophysiological role of the activin A–myostatin–follistatin system in modulating disease progression or response to therapy in patients with IIM; although, it has proved its importance in recent years. McConnell et al. showed also that follistatin is synthesized in many tissues usually induced by activin stimulation [119]. The circulating levels of activin A and follistatin may be associated with the inflammatory condition in IIM. The overproduction of follistatin in response to inflammation-induced muscle damage is presumed that could serve as a compensatory mechanism leading to lower levels of myostatin and its signaling attenuation [120].

In conclusion, follistatin can be modulated as a therapeutic factor in the IIM, inhibiting myostatin to maintain muscle mass at a normal level.

## 8. Decorin

There are very few studies regarding the proteoglycan decorin in skeletal muscle and even fewer regarding its presence in IIM. However, it is an important myokine that binds myostatin. It is released from contracting human myotubes, and its levels are increased in the response to acute resistance exercise and chronic training in humans. Decorin is part of the extracellular matrix [121, 122].

In an *in vivo* experimental study, it was shown that overexpression of decorin in murine skeletal muscle promoted expression of Mighty gene [123], negatively regulated by myostatin, and an increased response of myogenic factor Myod1 [124] and follistatin [112]. It is known that decorin has an antagonistic action with myostatin [125], which has been shown also on C2C12 myoblasts, decorin enhancing proliferation and differentiation of the cells by suppressing myostatin activity [126]. Another *in vitro* research reported that decorin binds to myostatin [127] reducing the myostatin inhibitory effect.

Goetsch et al. showed in an *in vit*ro study on C2C12 cells, which decorin binds TGF-*β*2, modifying the inhibition effect of this growth factor on cell migration and promoting the motility of myoblasts, positively influencing skeletal muscle regeneration [128]. Decorin has an antifibrotic effect also by forming the complex decorin/TGF-*β* [129, 130]. When decorin was injected into a traumatised muscle, *in vivo*, it could induce the regeneration of murine muscle, with minimal fibrotic scar tissue formation [131, 132].

Given the studies cited above and the fact that myostatin is upregulated in IBM, we can speculate that decorin may be downregulated in IIM. This myokine could be considered in the future a possible therapeutic target in myositis, in order to stimulate muscle regeneration.

## 9. Osteonectin

Osteonectin, named also SPARC (secreted protein acidic rich in cysteine), is a myokine that plays an important role in cell-matrix interactions, collagen binding, and bone mineralization. Osteonectin was found to be involved also in tumorigenesis inhibition by enhancing apoptosis in colon cancer cells [133]. It was observed an increased expression of SPARC in skeletal muscle after exercise, in mice and humans [133].

A study on muscular dystrophies such as Duchenne/Becker muscular dystrophy and congenital muscular dystrophy, and on IIM such as IBM and PM, have shown that SPARC is upregulated in muscle wasting [134].

In muscle progenitor cell line C2C12, osteonectin overexpression almost completely abolished myogenic differentiation [135]. Jorgensen et al. showed that SPARC-positive cells were present both in fetal and neonatal muscle [134]. Osteonectin protein was detected in mononuclear cells of which few were pax7 positive, in myotubes, and regenerating myofibers. The osteonectin expression-degree seemed to reflect the severity of the lesion. Primary human-derived satellite cells *in vitro* were found to express SPARC both during proliferation and differentiation.

Thus, it appears that SPARC plays a role in muscle cell regeneration. However, very limited studies are associated with the SPARC function in muscle development and pathology.

## 10. Insulin-Like 6

There is a small number of studies on Insl6. Insulin-like 6 (Insl6) is a myokine recently approached in myositis studies, being a member of insulin-like/relaxin family. It has been observed that Insl6 is overexpressed after the acute skeletal muscle injury [136].

Based on an experimental model of autoimmune myositis (EAM), induced in mice with human myosin-binding protein C, deficiency in Insl6 resulted in a worsened myositis phenotype with infiltration of CD4 and CD8 T-cells as well with an increased expression of inflammatory cytokines [137]. Muscle-specific Insl6 overexpression protects the muscle against the development of myositis and results in reduced lymphocyte infiltration in muscle tissue, decreased expression of inflammatory cytokines, and an improvement in motor function. The same study showed that an improvement in inflammatory conditions was produced *in vitro* by an acute hydrodynamic release of a plasmid encoding murine Isl6. The authors have established that Insl6 inhibits the proliferation and activation of T-cells. Furthermore, in a cohort of patients with PM and DM, Insl6 transcript expression in muscle was reduced. The authors of this study suggested that Insl6 could become a target for the treatment of myositis.

## 11. Myokines–MHC I–ER Stress—Proteasome Dialogue

An interesting toping regarding the complex connection between myokines and noninflammatory pathways implied in IIM has been attracting the attention of researchers in recent years.

Due to the ineffectiveness of the immunosuppressive treatment in some patients with myositis, research has turned in last years to new directions, such as the role of endoplasmic reticulum stress (ERs) in inflammatory pathology or, more recently, the role of myokines in maintaining this condition. The therapeutic approach in connection with these new features may lead to new more effective methods of treatment.

Regarding the role of ERs, many studies have been done and many significant results have emerged. Thus, permanent overexpression of MHC I in IIM can lead to ERs, with the accumulation of misfolded glycoproteins and activation of NF-*κ*B [138, 139]. Inversely, MHC I overexpression can be induced and sustained by ER stress, and this has been observed in both patients with myositis and in *in vivo* experimental studies on MHC I transgenic mice [140].

In order to move towards the role of myokines in the modulation of myositis pathology, we mention an *in vitro* study that demonstrated that overexpression of MHC I in C2C12 cell line myotubes led to the release of inflammatory chemokines CCL2 and CCL5 via the ER stress pathway [91] (Figure 1).

This nonimmune mechanism, ERs, can upregulate the expression of some cytokines as IL-1*β* and TNF-*α* [21], thus contributing to maintaining muscle weakness [141]. In sIBM, it was observed that ERs and myostatin deposition have been implicated in disease pathology [142]. Myostatin signaling pathway is an important target for the treatment of muscle atrophy. In Sachdev et al. study an analysis of the presence of myostatin precursors in a human muscle cell line was performed, and they found that increased levels of these precursors induce ERs. Metabolites of myostatin precursors were retained within the endoplasmic reticulum. Importantly to mention, ERs also impaired the secretion of mature myostatin. The authors speculate that reduced circulating myostatin growth factor could be one explanation for the poor clinical efficacy of drugs targeting the myostatin pathway in sIBM [142].

The complex pathway of protein degradation, from proteasome to endoplasmic reticulum, and the MHC I implication (Figure 1) was reviewed by Vigneron and Van den Eynde [143]. The ubiquitin-proteasome system (UPS) is the major ATP-dependent protein degradation system in cells, and it is considered essential to maintain cellular protein homeostasis and ensure the elimination of misfolded proteins [144, 145]. Proteasome-dependent proteolysis is also essential to regulate other cellular processes, such as cell differentiation, cell-cycle progression, or apoptosis. Proteins degraded by proteasome are ubiquitin tagged to be recognised by the components of proteasome. Upon proteasomal degradation, small peptides are further degraded by cytosolic peptidases to recycle the pool of amino acids. A fraction of the peptides released by proteasomal degradation system is transferred into the lumen of the endoplasmic reticulum where is further trimmed. Some of peptides associate then with MHC I and peptide-MHC I complexes are finally displayed at the cell surface for potential recognition by cytolytic T-lymphocytes which are major sentinels poised to rapidly recognize and destroy cells expressing mutant, infectious or tumoral proteins. Thus, the proteasome is a major regulator of protein homeostasis in cells.

An *in vitro* study on primary human muscle cell cultures from myositis patients showed that immunoproteasome is involved in the maintenance of myokines (IL-6, IL-1*β*, CXCL9, and CXCL10) production and in MHC I overexpression [16]. Its inhibition or administration of selective drugs increases the expression of myokines in myoblasts during inflammatory conditions. The immunoproteasome is actively upregulated in myofibers and responsible for MHC-I expression in IIM. But immunoproteasome has also nonimmune regulating function in inflammatory condition being involved in the degradation of inflammatory response mediators. The results of this study revealed that the expression of immunoproteasome is important to maintain the myokines homeostasis and myokines mediated attraction of immune cells in muscle tissue [16].

This complex system, once disrupted, can cause many cellular alterations in signaling pathways, and we can speculate that in the pathology of IIM these aspects must be investigated, especially if patients do not respond to normal anti-inflammatory treatment. Not only ERs, which is already a complicated system, intervene in these cases but also its interrelation with the proteasome and myokines can complicate the pathogenesis of myositis, so that in the future studies should look at this connection as well.

## 12. Conclusion

Analysing the expression of myokines in the pathological conditions of myositis, we realized that their careful modulation could contribute to the improvement of treatment for this group of diseases. After immunosuppressant therapy, especially in nonresponder patients, stimulating or inhibiting of the production of these muscle cytokines could be the missing link in therapy. As this group of diseases is heterogeneous, they have many determinants that are triggered by the autoimmune process, so the treatment should be complex and focus more than inflammation. Unfortunately, for now, the therapeutic options for patients with IIM are limited.

We have seen that some of myokines, as follistatin and Insl6, have a low expression in myositis, intense exercise being already a good modulator, so as to induce their overexpression for the benefit of muscle tissue. Most myokines are upregulated in IIM, as interleukins, chemokines, myostatin, and osteonectin. They could be modulated not only by exercise but also by blocking them in different ways as we have shown above.

The role of myokines in myositis is not yet fully elucidated, but it is obvious that the development of new drugs based on future studies will be beneficial and could contribute to a more effective therapy for patients with inflammatory myopathies.

## Figures and Tables

**Table 1:** Myokines present in idiopathic inflammatory myopathies. In this table, we present the main role and activity of the most important myokines in myositis.

Myokines	Role in myositis
IL-6	(i) Controversial: proinflammatory as cytokine, anti-inflammatory as myokine. (ii) High level in IIM patients and *in vitro* studies; harmful [46, 48]. (iii) Mediator of innate and adaptive immune response [27, 28]. (iv) Its production induced by inflammatory cytokines TNF-*α*, IL-17, IL-1*β* in myoblasts, *in vitro* [18, 46], not in IIM, but with possible same effects in IIM. (v) Blocking IL-6 receptors, positive effect, *in vivo* study on PM model [45]. (vi) Possible biomarker for DM patients [43].

IL-15	(i) Upregulated in myositis—muscle and serum (DM, PM patients) [61, 62]. (ii) Causes muscle weakness—DM, PM patients [64]. (iii) Closely connected with CD163 macrophages—PM patients and PM *in vitro* model [65]. (iv) Upregulates MMP-9 expression in PM [65]. (v) Possible promotor of autoimmune inflammation *in vivo* [67].

IL-18	(i) Implicated in autoimmune diseases [75]. (ii) Localised in inflammatory cells and capillaries in IIM patients [76]. (iii) High serum levels in DM/DM with interstitial lung disease [77, 78] and PM [77].

CXCL10	(i) High level in myositis. His CXCR3 receptor also—*in vivo* [85]; in sIBM patients [86]; in PM, sIBM, DM patients [87]; in juvenile DM [88]. (ii) Possible promotor of autoimmune inflammation through initiation and maintenance of type 1 T-helper cells [84]. (iii) Possible therapeutic target in adult myositis patients [90].

CCL2, CCL3, CCL4, CCL5	(i) MHC I overexpression leads to CCLs release, *in vitro* [91]. (ii) CCL2 increased levels in IIM patients [48, 82], including juvenile DM [92]. (iii) CCL3, CCL4—upregulated in IIM [93, 94]. (iv) CCL5—low expression in few inflammatory cells in IIM [93]. (v) CCR1 receptor—in macrophages and endothelial cells in sIBM [93]. (vi) CCR5 receptor—in inflammatory cells invading nonnecrotic muscle fibers in IIM [93]. (vii) CCL2 and CCR2 receptor, high levels in IIM patients [95]

CCL20	(i) Upregulated in the presence of IL-17 and IL-1*β* in muscle cells—*in vitro* study and in DM, PM muscular biopsies [45].

Myostatin	(i) Accumulates and associates with aggregates containing A*β* in sIBM patients [108]. (ii) Upregulated in IIM [109]. (iii) Myostatin gene expression attenuated, inhibitors gene expression (follistatin) upregulated in resistance training in IBM [110, 111].

Follistatin	(i) Upregulated in inflammatory diseases [120]. Proposed as an inhibitor of myostatin. (ii) Follistatin gene transfer to sIBM patients—clinical trial, resulted in an improvement in muscle regeneration [117]. (iii) Follistatin high serum levels concomitant with decreased serum myostatin levels in IIM patients [118].

Decorin	Although studies have been performed on its role as opposed to myostatin in skeletal muscle, there are no studies on IIM pathology regarding decorin.

Osteonectin	(i) Upregulated in IIM and in muscular dystrophies [134].

Insl6	(i) Protect the muscle against IIM development; downregulated in IIM—*in vivo* study [137]. (ii) Insl6 deficiency resulted in a worsened myositis phenotype—*in vivo* [137]. (iii) When it is downregulated, inflammatory cytokines expression is increased [137]. (iv) Inhibits the proliferation/activation of T-cells [137]. (v) Reduced in PM and DM patients (Insl6 transcript expression) [137].
